# Genetic Diversity and Genome Size Variability in the Russian Genebank Collection of Tea Plant [*Camellia sinensis* (L). O. Kuntze]

**DOI:** 10.3389/fpls.2021.800141

**Published:** 2022-02-02

**Authors:** Lidiia S. Samarina, Alexandra O. Matskiv, Ruset M. Shkhalakhova, Natalia G. Koninskaya, Magda-Viola Hanke, Henryk Flachowsky, Alexander N. Shumeev, Karina A. Manakhova, Lyudmila S. Malyukova, Shengrui Liu, Juanyan Zhu, Maya V. Gvasaliya, Valentina I. Malyarovskaya, Alexey V. Ryndin, Eduard K. Pchikhachev, Stefanie Reim

**Affiliations:** ^1^Federal Research Centre the Subtropical Scientific Centre of the Russian Academy of Sciences, Sochi, Russia; ^2^Federal Research Centre for Cultivated Plants, Institute for Breeding Research on Fruit Crops, Julius Kühn-Institute (JKI), Dresden, Germany; ^3^Center of Genetics and Life Science, Sirius University of Science and Technology, Sochi, Russia; ^4^State Key Laboratory of Tea Plant Biology and Utilization, Anhui Agricultural University, Anhui, China

**Keywords:** *Camellia sinensis*, genetic diversity, molecular markers, germplasm collection, ploidy level, flow cytometry, cold tolerance

## Abstract

The tea collection of the FRC SSC RAS (Sochi, Maykop in Russia) represents one of the northernmost germplasm comprising a number of locally derived cultivars and ɣ-irradiation mutants. The latter are often characterized by larger genome size, which may lead to better adaptation to biotic and abiotic stress. Such genotypes may be a valuable genetic resource for better adaptability to extreme environmental conditions, which could enable tea cultivation outside global growing regions. Microsatellite markers are often the best choice for genetic diversity analysis in genebank collections. However, their use in polyploid species is questionable because simple sequence repeat (SSR) allele dosage cannot be readily determined. Therefore, the efficiency of SSR and start codon targeted (SCoT) markers was investigated using 43 selected cultivars from the Russian genebank collection derived from mutant breeding and clonal selection. Previously, the increase in genome size was confirmed in 18 mutants within this collection. Despite the presence of polyploid tea genotypes, our study revealed higher efficiency of SSR markers than SCoT markers. Subsequent SSR analysis of the 106 genotypes in the Russian genebank collection revealed three distinct genetic clusters after STRUCTURE analysis. Greater genetic variation was observed within genetic clusters than between clusters, indicating low genetic variation between collections. Nevertheless, the northernmost tea collection exhibited a greater genetic distance from the other two clusters than they did from each other. Close genetic relationships were found between many cultivars with particularly large leaves and mutant forms. Pearson’s correlation analysis revealed a significant, moderate correlation between genome size and leaf area size. Our study shows that microsatellite fingerprinting is useful to estimate the genetic diversity and genetic background of tea germplasm in Russia despite polyploid tea accessions. Thus, the results of our study contribute to the development of future tea germplasm conservation strategies and modern tea breeding programs.

## Introduction

Germplasm collections outside of the main growing regions are an important source of genetic diversity for crop improvement. Such border growing regions can be useful for providing new genetic resources with a wider genetic base and a higher adaptability to changing environmental conditions. Local genotypes and landraces in such border regions offer a broad range of resilience to different agro-climatic conditions and are therefore an important resource for breeding purposes. Therefore, local genotypes should be characterized and conserved for appropriate utilization (Beris et al., 2016; Tan et al., 2018; Seyis et al., 2019; Maharramov et al., 2020; Zhang et al., 2020). Furthermore, clarifying the genetic diversity and genetic structure of genebank collections is important for understanding domestication mechanisms and developing efficient breeding strategies for horticultural crops (Zhao et al., 2014; Wambulwa et al., 2016; Niu et al., 2019).

The northwestern Caucasus in Russia (Krasnodar region, Sochi) and Adygea republic (Maykop) is one of the northernmost border regions of commercial tea cultivation in the world. Domestication of the tea plant in Caucasus started about 150 years ago in Georgia (41°55′37″N 42°00′02″E). In the 19th century, seeds of tea plants were introduced from China, Japan, India, Sri Lanka, and Indonesia, representing a great genetic diversity of hybrids. From Georgia, cultivation spread to the south (Turkey), but also to the northern region in Maykop (Adygea Republic, Russia) (44°36.5858′0″N, 40°6.031′0″E) (Gvasaliya, 2015). Tea plant [*Camellia sinensis* (L.) Kuntze] is one of the important perennial crops, whose cultivated area and world production have increased twofold in the last 5 years. According to the classification system of Ming (2000), the cultivated tea plant is currently divided in two varieties, namely *C. sinensis* var. *sinensis* (CSS) and *C. sinensis* var. *assamica* (CSA). CSS is the Chinese variety that has small leaves and can tolerate short frosts. CSA originates from the Assam region of northern India, has larger leaves, and is less tolerant of cold weather. It is believed that only CSS and hybrid CSS × CSA genotypes were successfully domesticated in our region. The different morphological and biochemical characteristics of CSS and CSA are mainly due to their different geographical distribution and growing environment (Meegahakumbura et al., 2016; Hao et al., 2018; Zhang W. et al., 2018; Li et al., 2019; An et al., 2020; Wang et al., 2020).

To increase the genetic diversity of the local tea germplasm in the Caucasus and to develop new elite cultivars with valuable horticultural traits, breeding activities such as chemical mutagenesis and ɣ-irradiation, clonal selection, and controlled hybridization were conducted. As a result, a number of elite genotypes with a wide range of phenotypic variability have been developed. In particular, the mutant tea tree varieties can be of great benefit to growers as they are often polyploid, resulting in superiority in vegetative growth, higher yield, and improved resistance compared to diploids.

The market value of tea is largely dependent on the tea plant species and the cultivars used. Therefore, it is important to provide efficient DNA markers that can be used to identify tea varieties, regardless of their ploidy level, geographic origin, and the environmental conditions (Kalia et al., 2011; Yang and Liang, 2014).

Several molecular markers for tea plants are available (Niu et al., 2019; Xia et al., 2020). Based on the recently published genome assemblies (Wei et al., 2018) and tea genome database (Xia et al., 2019), many locus-specific microsatellite markers were developed (Liu et al., 2017, 2018). In general, microsatellite markers are the most valuable tools for characterization of plant genetic resources or population genetic analysis. However, for polyploid individuals, such as possibly the mutant tea accessions, the usage of SSRs are doubtful due to the difficulties in determining allele dosage (Pfeiffer et al., 2011; Trapnell et al., 2011). Although dominant markers have the disadvantage of not providing direct information on heterozygosity, their use can help avoid problems with dosage uncertainty when working with polyploids (Dufresne et al., 2014). Therefore, dominant markers such as start codon targeted (SCoT) markers can provide further reliable information on genetic diversity (Gorji et al., 2011; Pakseresht et al., 2013; Xanthopoulou et al., 2015; Etminan et al., 2016, 2018). SCoTs are based on polymorphism in the short, conserved region in plant genes surrounding the ATG translation initiation codon. They amplify regions between genes, which may even allow the identification of associations with relevant traits. In recent studies, SCoT and SCAR markers were used to study the genetic diversity in tea and barcoding of important genotypes (Xu et al., 2019; Chaeikar et al., 2020). In addition to molecular analysis, genome size determination is also an important tool for characterizing germplasm to understand taxonomic relationships and track evolutionary changes and domestication events (Sharma et al., 2019).

To date, there are no investigations on the genetic background of the Russian cultivar collection and their genetic relationships. The wide range of phenotypic variability of tea in the Western Caucasus makes it difficult to clarify the origin and the relationships of these collections based on morphological characteristics. Therefore, the genetic relationships between the 106 germplasm accessions of the Russian tea collection of FRC SSC RAS, which is located close to the black sea coast in Sochi (Russia), was genetically investigated. For this purpose, SSR markers on eight different linkage groups were selected. Flow cytometry was used to determine the ploidy level in 43 selected cultivars resulting from mutation and clonal selection within the collection. In addition, these selected genotypes were analyzed with SCoT markers to compare the efficiency of SSR and SCoT markers in characterizing polyploid tea accessions. The study aimed to: (I) estimate the genetic diversity, genetic structure, and relationships among genebank accessions using SSR markers; (II) investigate intraspecific genome size variability of selected cultivars derived from mutant breeding and clonal selection; (III) evaluate the efficiency of the markers used for the molecular characterization of polyploidy tea accessions; and (IV) evaluate correspondence between molecular marker data and phenotypic data. The results will lead to a better understanding of the genetic background of the tea germplasm in Russia and contribute to the development of modern tea breeding programs and future tea germplasm conservation strategies.

## Materials and Methods

### Plant Material and Phenotyping

The plant material was obtained from the field gene bank of the Federal Research Centre the Subtropical Scientific Centre of the Russian Academy of Sciences (FRC SSC RAS, Supplementary Table 1). The genebank collection contains 31 mutant forms derived in USSR between 1970 and 1980 by γ-irradiation of seeds (mostly cv. “Kolkhida,” cv. “Qimen”). Twelve important cultivars were included, namely, “Kolkhida,” “Karatum,” “Sochi,” “Kubanskii,” “Qimen,” “Gruzinskkii7,” “Gruzinskii8,” “Gruzinskii15,” “Gruzinskii79-79,” “Krasnodarskii1,” “Krasnodarskii2,” and “Krasnodarskii3.” In addition, the frost-tolerant breeding lines “Adygeiskii1-5,” “Zmeyka,” “Qimen-pH-tolerant1-3,” “Leftraw,” and “GP” (Georgian population) from the northern field plots (Tuapse, Maykop) were included. Furthermore, locally derived breeding lines and hybrids were studied (Supplementary Table 1). All plants were about 31–33 years old and clonally propagated with 10–30 replicates per cultivar on the experimental field collection of FRC SSC RAS (in Sochi, Tuapse and Maykop). Phenotypic evaluation was performed for 43 accessions (27 mutant forms, 12 clonal selections, and 4 cultivars) of the collection in the period 2018–2021 using IPGRI^1^ descriptors. The following leaf parameters were evaluated during these years: length, width, area (cm^2^), length to width ratio, petiole length (mm), surface, edge, base, leaf angle, leaf denticles, denticles depth, and leaf texture (Supplementary Table 1). The leaf-related traits were characterized using ten most fully expanded mature leaves collected from each cultivar and each replicate. The leaf area size was classified for all 106 genotypes of the entire tea collection according to Wang and Tang (2012): (1) small-leaf (leaf area ≤ 20 cm^2^); (2) middle-leaf (leaf area 20 – 40 cm^2^); (3) large leaf (leaf area 40 – 60 cm^2^); and (4) extra-large leaf (leaf area ≥ 60 cm^2^).

### Genome Size Evaluation

Flow cytometry was used to analyze the genome size of 27 mutant tea and 12 clonal selections, including the diploid standard cultivars “Sochi” and “Kolkhida,” the aneuploidy cultivar “Karatum,” and the triploid cultivar “Kubanskii.” In each flow cytometry analysis, the cultivar “Kolkhida” was used as reference standard for plant DNA content determination. The chromosome number and genome size of “Kolkhida” was previously determined (Efremov et al., 2018; Gvasaliya and Samarina, 2019). Pieces of 2 cm^2^ young leaves were collected and chopped in 1 ml of ice-cold nuclei isolation buffer with a sharp razor blade. For screening purposes, the leaves from different genotypes were chopped separately. For precise genome size estimation, Kolkhida’s leaves were chopped together with the sample of interest in the same buffer. WPB buffer was used as the nuclei isolation buffer since its components reduced the effects of phenolic compounds and preserved chromatin integrity (Doležel et al., 2007; Huang et al., 2013). The WPB buffer was prepared as follows: 0.2 M TrisHCl, 4 mM MgCl_2_x6H_2_O, 2 mM EDTA Na_2_x2H_2_O, 86 mM NaCl, 10 mM sodium metabisulfite, 1% PVP-40, 1% (v/v) Triton X-100, pH 7.5. The isolated nuclear suspension was filtered through a 40 μm nylon mesh, supplemented with RNAse (50 μg/ml) and propidium iodide (50 μg/ml) and incubated on ice for 2 h before the analysis.

Nuclear suspensions were acquired with a Cytoflex flow cytometer and then analyzed using FlowJo™ v10.8 Software (BD Life Sciences). The PI fluorescence was exited with a 488 nm diode laser and collected with a 610/20 filter. The samples were acquired at a slow flow rate (10 μl/min) that corresponded to ∼100 nuclei/sec, and at least 10,000 nuclei were acquired for each sample. Medians of fluorescence intensity of G0/G1 (2C) peaks were used for calculations of the DNA content. Results with a coefficient of variation (CV) of G0/G1 less or equal 5% and a CV of G2/G1 2.00 ± 0.05 were considered as reliable. The absolute amount of DNA content was calculated using a linear relationship between the ratio of 2C value peak of the respective sample and the internal standards *C. sinensis* cv. “‘Kolkhida”:2C DNA content = 6.99 pg (Efremov et al., 2018) and *C. sinensis* cv. “Sochi,” 2C DNA content = 6.95 pg (Hembree et al., 2019). For each accession, three independent replicates from different plants were analyzed separately.

### DNA Extraction and Genetic Diversity Analysis

Young and healthy leaves of each accession were collected in 2 ml tubes and dried using silica gel. The leaf material was stored at 4°C until DNA isolation. The dried leaf material was ground in a Mixer mill apparatus (Retsch, Germany), and DNA isolation followed immediately using the DNeasy Plant Mini Kit (Qiagen, Germany) according to the manufacturer’s instructions. All samples were diluted to 10 ng μl^–1^ and stored at −20^o^C.

For SSR analysis of the 106 accessions of the tea collection, eight nuclear microsatellite (SSR) primer pairs (Wang et al., 2016) were combined in two multiplexes with four primers in each multiplex (Supplementary Table 2). The forward primers in one multiplex reaction were labeled with four different dyes (ATTO565, ATTO550, ATTO532, and 6-FAM, Eurofins, Germany). The multiplex PCRs were carried out following the manufactures guide of the “type-it microsatellite kit”^®^ (Qiagen, Germany). The multiplex electrophoresis was performed on a 3500xl DNA Analyzer (Applied Biosystems, Foster City, United States) at the Julius Kühn-Institut (JKI), Institute for Breeding Research on Fruit Crops, Dresden, Germany.

For SCoT analysis 43 accessions, derived from mutant breeding and clonal selection, were analyzed with 36 SCoT primers (Collard and Mackill, 2009) (Supplementary Table 2). The SCoT PCR reaction mixture consisted of 10 μl 2x HS-TaqPCR reaction buffer including Hot Start Taq-Polymerase (Biolabmix, Russia), 0.4 μl of primer (10μM), 2 μl of DNA (20 ng μl^–1^), and DEPC-treated water in a total PCR volume of 20 μl. Amplification was carried out in the MiniAmp thermal cycler (Thermo Fisher Scientific, United States) with the following program: primary denaturation 5 min at 95°C, 35 cycles with denaturation at 95°C for 1 min, annealing at 52°C for 1 min, elongation at 72°C for 2 min, and final elongation at 72°C for 5 min. The separation of SCoT-fragments was performed in a 2% agarose gel for 2.5 h at 90 V in 1 × TAE buffer.

### Statistical Analysis

To compare the efficiency of SSR and SCoT makers, genetic diversity parameters were calculated based on the selected 43 accession derived from mutant breeding and clonal selection. For the SSR dataset, this was done using the software program GeneAlex ver. 6.5 (Peakall and Smouse, 2006, 2012) with mean number of alleles by locus (*Na*) and effective number of alleles (*Ne*), Shannon’s information index (*I*) expected heterozygosity (*He*), observed heterozygosity (*Ho*), and number of private alleles. For SCoT data analysis, the online resource^2^ was used to estimate following parameters: *P* = polymorphic loci (%); *I* = Shannon’s information index; and genetic diversity (*H*) (Amiryousefi et al., 2018).

The analysis function “Matches” in GeneAlex ver. 6.5 (Peakall and Smouse, 2006, 2012) was used to identify genotypes with identical allelic patterns within SSR and SCoT dataset. To estimate the number of marker combinations necessary to discriminate all genotypes, the probability of identity (PI) was calculated with GenAlex ver 6.5. Based on the probability of identity, the power of discrimination (*PD*) for each marker was calculated as (PD = 1 − PI). The *PD* indicates to which probability the marker can discriminate the genotypes in the entire data set.

Additionally, principal coordinate analysis (PCoA) was performed to visualize the genetic distance of the accessions based on SSR and SCoT markers, respectively. GeneAlex ver. 6.5. was also used to calculate the Mantel correlation between the SSR and SCoT matrices.

Genetic diversity parameters of the 106 accessions in the genebank collection were calculated for each SSR locus as described above. To verify the genetic structure within the 106 accessions of the tea genebank collection, the model-based clustering method was applied using the software STRUCTURE ver. 2.3.4. (Pritchard et al., 2000). The parameters were 50,000 burn-in periods and 50,000 Markov Chain Monte Carlo repetitions using the admixture model with correlated allele models. The software program STRUCTURE HARVESTER (Earl and Vonholdt, 2012) was used for detecting the most likely value for *K* based on Evanno’s ΔK method (Evanno et al., 2005). Subsequently, a phylogenetic tree was drawn based on the dissimilarity matrix of the SSR data using DARWIN ver.6. Using the software program SAS ver. 9.4., Pearson’s correlation was analyzed between the 2C DNA content and the leaf area size (cm^2^).

## Results

### Genome Size Variability and Phenotypic Evaluation of Accessions Derived From Mutant Breeding and Clonal Selection

Flow cytometry analysis of 43 samples with the included internal controls (“Kolkhida” and “Sochi”) revealed separate position of G0/G1peaks of the tested and control samples. The DNA content in the collection varied from 2C = 5.69 pg (in #502) to 2C = 12.71 pg (in #619). The DNA content of the both diploid standard cultivars “Sochi” and “Kolkhida” was 7.0 pg (Table 1). Eighteen mutant genotypes showed a remarkably higher 2C DNA content as the 2*n* cultivars “Kolkhida” and “Sochi.” The increased DNA content of 7.4–12.7 pg was observed in the 18 mutant forms related to aneuploids (2C = 7.6 ± 0.2 pg: #53, #62, #2264, “Karatum,” #2697, #1484, #1467), triploids (2C = 8.3 ± 0.5 pg: #321, #1102, #1292, #3509, #212, #501, #50, #56, #1326, “Kubanskii”) and tetraploids (tetraploid (2C = 12.7 pg: #619) (Table 1).

**TABLE 1:**
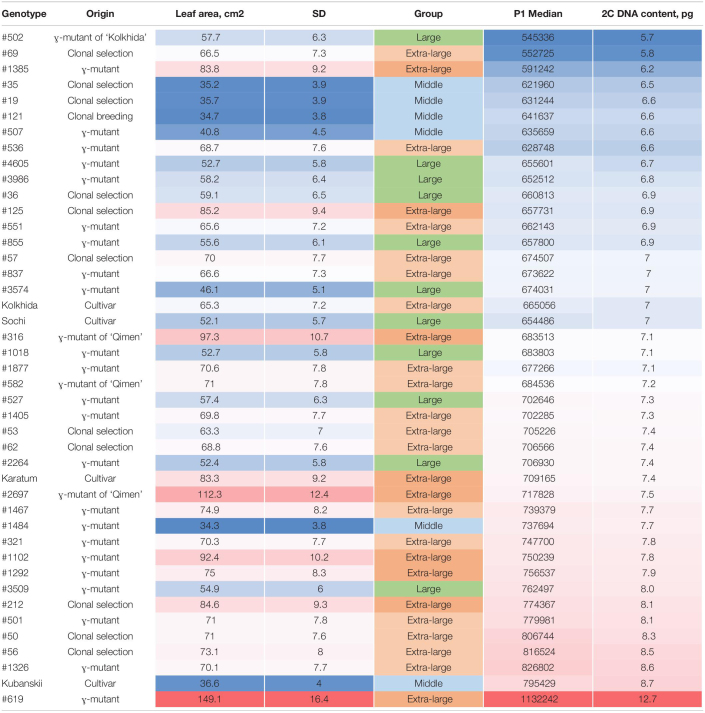
Nuclear DNA content (pg/2C mean) of tea accessions ordered by the genome size from low (blue) to high (red).

*SD, standard deviation.*

**FIGURE 1:**
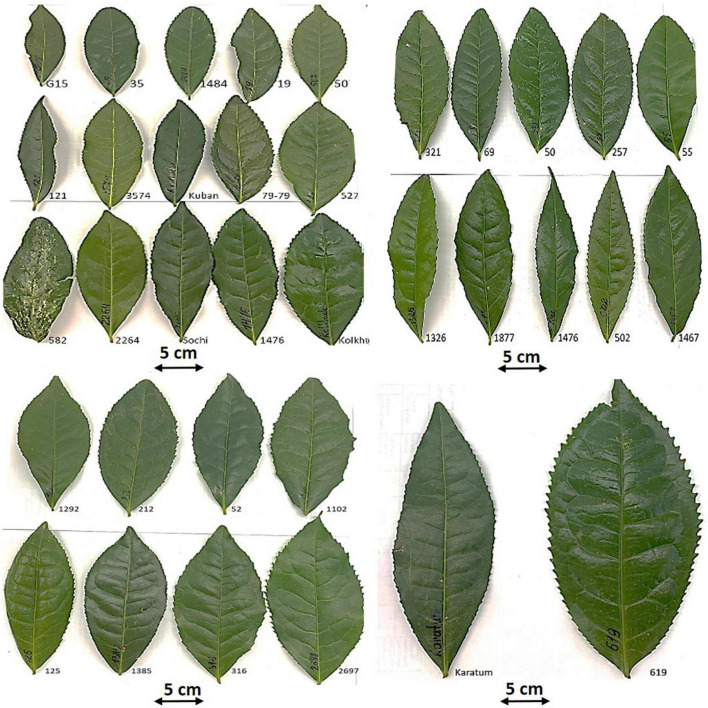
Morphological variability of the typical mature leaves of the mutant forms and cultivars of tea collection in FRC SSC RAS, Sochi, Russia.

High level of phenotypic variability was observed amongst the 43 studied individuals (Figure 1 and Supplementary Table 1). The average leaf length in the genebank collection was 13.9 cm and varied from 9.8 to 19.7 cm, the average leaf width was 6.1 cm and varied from 3.9 to 9.7 cm. The average leaf area size of the accessions was 66.8 cm^2^ and varied from 34.3 cm^2^ to 149.1 cm^2^. In all cases, the mutant accession #619 had the largest leaves. No small-leaf genotypes (leaf area size < 20 cm^2^) were detected in the collection and only 14% of the genotypes had a middle leaf area size (20–40 cm^2^). In contrast 26% of the accessions were attributed to the large-leaf group (40–60 cm^2^), whereas the main part of the accessions (60%) were even attributed to the extra-large group (leaf area size > 60 cm^2^) with a range of the leaf area from 63.3 to 149.1 cm^2^ (Table 2, Figure 1, and Supplementary Table 1).

**TABLE 2:** Leaf area variability in the collection of tea plant 43 accessions derived from mutant breeding and clonal selection in FRC SSC RAS, Sochi, Russia.

Group	Leaf area, cm^2^	No of accessions	Accession label
Group (1) small leaf area	<20	–	–
Group (2) middle leaf area	20 – 40	6	#1484; #121; #35; #19; #507; “Kubanskii”
Group (3) large leaf area	41 – 60	11	#36; #502; #527, #855, #1018; #2264; #3509; #3574; #3986; #4605; “Sochi”
Group (4) extra-large leaf area	>60	26	#50; #53; #56; #57; #62; #69; #125; #212; #316; #321; #501; #536; #551; #582; #619; #837; #1102; #1292; #1326; #1385; #1405; #1467; #1877; #2697; “Karatum”; “Kolkhida”
Total		43	

Most of the cultivars (68%) with extra-large leaf area (>60 cm^2^) showed an increased genome size, namely, #619, “Karatum,” #62; #1405; #2697; #1326; #321; #501; #50; #1467; #1292; #1385; #212; #56; #1102. Most of the middle-leaf genotypes showed diploid genome size with two exceptions (#1484 and “Kubanskii”; Table 1). Pearson’s correlation analysis revealed a significant moderate correlation between the 2C DNA content and the leaf area size (*r* = 0.57, *p* < 0.0001).

### Efficiency of Simple Sequence Repeat and Start Codon Targeted Markers in Accessions Derived From Mutant Breeding and Clonal Selection

The efficiency of SSRs and SCoT marker were evaluated selecting 43 tea accessions that derived from mutant breeding and clonal selection. Of these, 18 accessions with increased ploidy were identified.

Eight SSR markers of different linkage groups (LG) were tested. Of these, seven markers TM447 (LG01), TM514 (LG02), TM337 (LG03), TM589 (LG05), TM341 (LG06), TM415 (LG07), and TM352 (LG08) showed clear polymorphism and were included in the study (Table 3). The average number of different alleles per locus was *Na* = 4.79, ranging from *Na* = 2.88 for the cultivars to *Na* = 7.25 for the mutants (Table 3). The average effective number of alleles was *Ne* = 2.58 and showed only slight differences between the accessions derived of clonal selection or mutant breeding compared to the cultivar accessions. The mean expected heterozygosity was *He* = 0.53, with the lowest value for the cultivars (*He* = 0.48) and the highest value for the mutants (*He* = 0.56). Private alleles (with a frequency > 0.05) were found in all the groups (Table 3).

**TABLE 3:** Genetic diversity parameters calculated for 43 accessions derived from mutant breeding and clonal selection based on 7 SSR markers.

Pop	*N*	*Na*	*Ne*	*I*	*Ho*	*He*	*PA*
Clonal selection	12	4.25	2.61	1.04	0.44	0.55	4
ɣ-mutant	27	7.25	2.72	1.19	0.36	0.56	6
Cultivar	4	2.88	2.42	0.84	0.47	0.48	1
Mean	14	4.79	2.58	1.02	0.42	0.53	
SD	2	0.55	0.23	0.10	0.06	0.05	

Na, no. of different alleles; Ne, no. of effective alleles = 1/(Sum pi^∧^2); I, Shannon’s Information Index = −1* Sum [pi * Ln (pi)]; Ho, observed heterozygosity = No. of Hets/N; He, expected heterozygosity = 1 – Sum pi^∧^2; uHe, unbiased expected heterozygosity = [2N/(2N-1)] * He; F, fixation index = (He – Ho)/He = 1 – (Ho/He); where pi is the frequency of the ith allele for the population & sum pi^∧2^ is the sum of the squared population allele frequencies. PA = number of private alleles with a frequency > 0.05.

Out of 36 SCoT primers, 25 primers (SCoT1, 2, 9, 10, 11, 13–16, 18–24, 27–32, 34–36) showed low amplification quality with weak or fuzzy bands and were removed from the further analysis. The remaining eleven SCoT primers (SCoT3, SCoT4, SCoT5, SCoT6, SCoT7, SCoT8, SCoT12, SCoT17, SCoT25, SCoT26, and SCoT33) showed reproducible results with clear polymorphisms and resolution within tea genotypes. Using these eleven SCoTs with 43 accessions, a total of 175 bands were detected resulting in 67% polymorphism. The average number of different alleles among the three groups was 1.58 and ranged from *Na* = 1.27 for the cultivars to *Na* = 1.82 for the mutants (Table 4).

**TABLE 4:** Genetic diversity parameters calculated for 43 accessions derived from mutant breeding and clonal selection based on 11 SCoT markers.

Pop	*N*	*Na*	*Ne*	*I*	*Hh*	*% P*
Clonal selection	12	1.64	1.44	0.38	0.26	72.7%
ɣ-mutant	27	1.82	1.40	0.38	0.25	81.8%
cultivar	4	1.27	1.35	0.28	0.19	45.5%
Mean	14	1.58	1.40	0.35	0.23	66.7%
SD	2	0.12	0.06	0.05	0.03	10.9%

Na, no. of different alleles; Ne, no. of effective alleles = 1/(Sum pi^∧^2); I, Shannon’s Information Index = −1* Sum [pi * Ln (pi)]; h, Diversity = 1 – (p^∧^2 + q^∧^2); % P, percentage of polymorphic loci.

The power of discrimination was high for most SSRs (mean *PD* = 0.74) and ranged from *PD* = 0.21 (TM514) to *PD* = 0.95 (TM589) for the 43 accessions. For the SCoT markers, the power of discrimination was slightly lower (mean *PD* = 0.71) and ranged from *PD* = 0.67 (SCoT33) to *PD* = 0.74 (SCoT17) (Table 5). Based on the SSR markers, no identical DNA-fingerprints were observed among the 43 accessions with the “matches” function in GenAlex. The combination of the 5 SSRs with the highest *PD* values were sufficient to discriminate the genotypes in the data set (data not shown). This was different after running the “matches” function in GenAlex again based on the SCoT markers. Although the *PD* value for the SCoT marker was only slightly lower than for the SSR markers, it was not possible to distinguish all 43 accessions based on the 11 SCoT markers. Only 20 individuals had different allele patterns, while the remaining 23 genotypes were grouped into 10 clonal groups of 2–4 genotypes with identical allele patterns.

**TABLE 5:** Discrimination power (*DP*) of the 8 SSR markers and 11 SCoT markers for 43 tea accessions that derived from mutant breeding and clonal selection.

	Pop	Clonal selection	ɣ-mutant	Cultivar	Total
	** N**	**12**	**27**	**4**	**43**
SSR	TM337	0.87	0.70	0.83	0.80
	TM343	0.75	0.77	0.00	0.75
	TM447	0.76	0.92	0.72	0.90
	TM514	0.27	0.20	0.00	0.21
	TM341	0.66	0.72	0.84	0.73
	TM352	0.80	0.73	0.63	0.76
	TM415	0.64	0.79	0.87	0.82
	TM589	0.94	0.94	0.87	0.95
	** Mean**	**0.71**	**0.72**	**0.59**	**0.74**
SCoT	SCoT3	0.65	0.76	0.69	0.72
	SCoT4	0.76	0.77	0.78	0.77
	SCoT5	0.67	0.67	0.73	0.68
	SCoT6	0.67	0.73	0.71	0.71
	SCoT7	0.69	0.69	0.74	0.69
	SCoT8	0.60	0.68	0.64	0.65
	SCoT12	0.72	0.71	0.67	0.71
	SCoT17	0.74	0.74	0.72	0.74
	SCoT25	0.74	0.65	0.73	0.68
	SCoT26	0.80	0.74	0.77	0.76
	SCoT33	0.68	0.64	0.80	0.67
	**Mean**	**0.71**	**0.71**	**0.72**	**0.71**

*The total number of samples (N) and the mean values (MEAN) are indicated in bold.*

### Comparing Genetic Distances Among Tea Genotypes Based on Simple Sequence Repeat and Start Codon Targeted Markers

**FIGURE 2:**
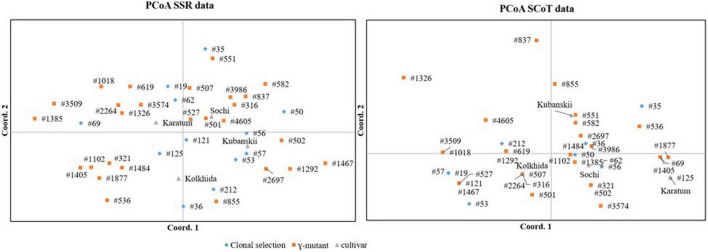
PCoA for 43 tea accessions that derived from mutant breeding and clonal selection based on SSR (left) and SCoT (right) data.

To visualize the similarities of the SCoT and SSR data, PCoA was performed for the 43 genotypes separately for each data set. According to the results of the “matches” function based on SCoT markers, several accessions showed no genetic distances between each other (e.g., “Karatum” and #125; “Kolkhida” and #507, #316 and #2264; Figure 2). The PCoA based on the SSR showed a divergent result in which the cultivars showed a more distant relationship. This observation was confirmed by the Mantel test, which showed no correlation between the SCoT and SSR data matrices (*Rxy* = −0.0733).

### Genetic Diversity, Genetic Structure, and Relationships in Tea Germplasm Collection Based on Simple Sequence Repeat Genetic Data

Since the efficiency of selected SSR markers was higher than for the SCoT markers, the entire tea germplasm collection was analyzed only with the seven SSR markers (TM337, TM447, TM514, TM341, TM352, TM415, and TM589). The average number of different alleles per locus was *Na* = 10.7, ranging from *Na* = 6.0 for TM352 to *Na* = 13.0 for TM337 and TM589 (Table 3). The average effective number of alleles was *Ne* = 3.3, ranging from *Ne* = 1.13 for TM514 to *Ne* = 6.17 for TM589. The mean expected heterozygosity was *He* = 0.6, with the lowest value for TM514 (*He* = 0.11) and the highest value for TM589 (*He* = 0.84).

**FIGURE 3:**
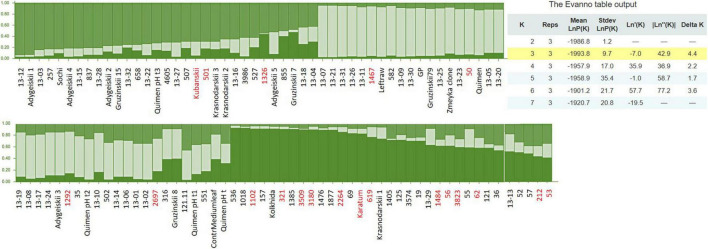
Genetic structure of the 106 tea accessions assessed by 7 SSR markers. Red labels indicated the genotypes with increased genome size, comparing to control cv. “Kolkhida” and cv. “Sochi.”

Following STRUCTURE analysis, 106 accessions were divided into the three genetic clusters (Figure 3). The first cluster combined 31 accessions, and the most northerly grown accessions joined this group. The second cluster consisted of 41 accessions. Most of them (59%) were hybrids, probably obtained from Quimen landraces. Several mutant forms and clonal varieties also joined this group, namely #1467, #582, #50, #35, #502, #316, #2697, and #551. The third cluster combined 34 accessions, which are mostly the big-leaf mutant forms with increased genome size (red labels, Figure 3). High levels of genetic admixture was observed in each of three genetic clusters. The suspected “Quimen”/“Kolkhida” origin of many mutant forms was confirmed by STRUCTURE analysis. The mutants #1467, #582, #502, #316, #2697, and #551, which are included in cluster 2 along with the Quimen landraces, indicate that these genotypes were derived from “Quimen” seeds. In contrast, the mutants #1018, #536, #1102, #157, #321, #1385, #3509, #3180, #1476, #1877, #2264, #69, and #619 were grouped together with the cultivar “Kolkhida” in cluster 3, indicating that these genotypes were derived from “Kolkhida” seeds.

**FIGURE 4:**
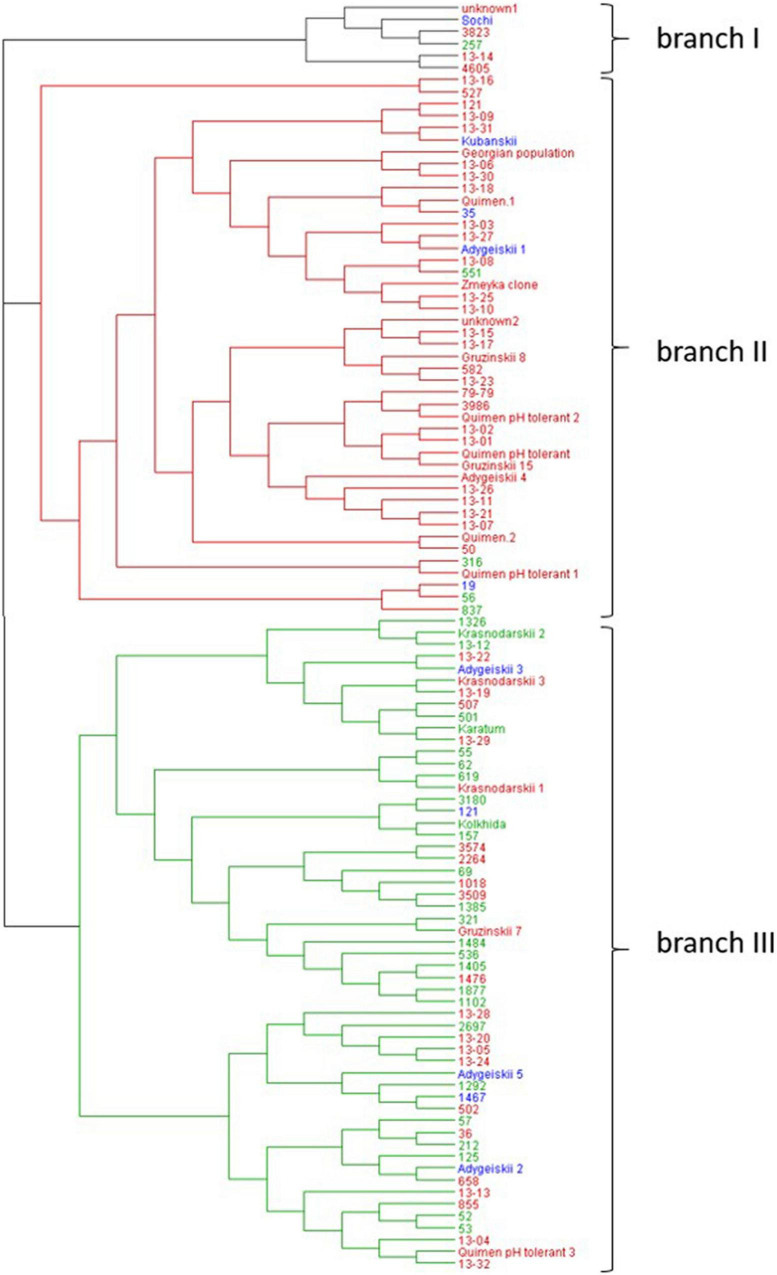
Neighbor-joining phylogenetic tree based on the seven SSR markers for the 106 tea accessions using Nei’s genetic distances. Blue letters: genotypes with middle size leaves (20 – 40 cm^2^), red letters: genotypes with large leaves (41–60 cm^2^); and green letters: genotypes with extra-large leaves (>60 cm^2^).

The subsequent neighbor joining analysis based on SSR data yielded three main branches in the collection of 106 accessions (Figure 4). Each branch included mainly accessions with similar leaf area sizes, indicating that this phenotypic trait is associated with the genetic relationship of the accessions (Supplementary Table 1). Branch I combined six accessions including the cultivar “Sochi,” clones and mutants. With exception of #257, the accessions in this branch have middle- or large leaf sizes up to 60 cm^2^.

Branch II combined 45 accessions that were mainly hybrid forms as well as clonal selections of the four important local cultivars, namely “Kubanskii,” “Quimen,” and “Adygeiskii,” “Gruzinskii8,” “Gruzinskii15,” “Gruzinskii79.” Most of these accessions in this branch also belong to the group with middle- or large leaf area size up to 60 cm^2,^ with the exception of four genotypes, namely #551, #316, #56, and #837 (indicated in green).

Branch III contained 55 genotypes of mainly mutant forms. A main part of the genotypes in this group was characterized by increased genome size, and 26 genotypes showed extra-large leaf size greater than >60 cm^2^. Twenty-four genotypes were characterized by large leaves (indicated in red), whereas only 5 genotypes showed middle-size leaves (“Adygeiskii3,” “Adygeiskii5,” “Adygeiskii2,” #1467and #121).

## Discussion

Tea collection in Russia represents cold-tolerant tea varieties grown in Western and Northern Caucasus, where minimum winter temperature decreases to −12^o^C and −23^o^C, respectively. Controlled hybridization, γ-irradiation, and clonal selection have been applied as the main breeding tools to develop local genebank collection of tea plant. In this study, we analyzed genome size and phenotypical variability of 39 selected clonal and mutant accessions along with four important local cultivars. Furthermore, this selection was used to estimate the efficiency of selected SSR and SCoT markers. Subsequently genetic diversity, structure, and genetic relationships within the collection of 106 locally propagated tea genotypes were investigated.

### Genome Size and Leaf Area Size Variability in the Tea Collection

Ploidy and genome size can influence reproductive compatibility, fertility, and heritability of traits wherefore these data can be useful for understanding breeding potential in collections (Hembree et al., 2019). Our results showed remarkable intraspecific variation in the genome size of the 43 selected genotypes. Particularly, most of the mutant forms derived from “Kolkhida” or “Quimen” seeds treated with ɣ-irradiation showed increased genome size. Our results are consistent with other published data on tea, which indicated 2C DNA content in CSS ranged from 6.0–7.0 pg, whereas 4C DNA content was around 13.0 pg (Hembree et al., 2019). In CSA, the 2C DNA content ranged from 5.8–7.7 pg (Huang et al., 2013; Sharma et al., 2019). These data support our hypothesis on tetraploid nature of #619 and triploidy nature of several mutant forms, namely #321, #1102, #1292, #3509, #212, #501, #50, #56, and #1326. This genome size variation observed in mutant forms can be a result of insertions/deletions in DNA sequences or chromosome number alterations caused by ɣ-irradiation. This is particularly striking in species with large geographic distributions that likely exhibit high genetic differentiation as a result of adaption to extreme environmental conditions (Huang et al., 2013; Sharma et al., 2019). The limitation of our study is that we did not confirm the chromosome numbers of all our accessions due to their technical complexity.

In addition to the remarkable variation in genome size, we observed a wide range of morphological leaf traits in the 43 selected clonal, mutant accessions, and local cultivars. This reflects the complicated genetic background due to the different origins and breeding strategies of the accessions in the Russian collection. Among the different morphological leaf traits, the leaf area size (cm^2^) is one of the most important traits to characterize cultivar types and estimate the yield of tea plant (Rajkumar et al., 2010). Our study indicated that most of the cultivars (68%) with extra-large leaf area (>60 cm^2^) showed an increased genome size. Polyploids often are expected to contribute to larger plant size and higher yield than their diploid counterparts (Zakir and Dawid, 2020). Furthermore, polyploidy may induce a higher degree of resistance to biotic and abiotic stresses in existing tea cultivars, without causing changes in the desired parts of the genome (Zakir and Dawid, 2020). Alam et al. (2015) reported that triploids tea plants are more vigorous, hardier, and cold-tolerant than diploids.

Nevertheless, genome size is not always the main factor affecting leaf size, as shown by the moderate correlation between 2C DNA content and leaf size in our study. The varieties themselves are also characterized by different leaf sizes. Many publications indicated that most CSA varieties have thin big leaves [average leaf area of 54.8 cm^2^ (Rajkumar et al., 2010)]. In contrast, CSS varieties are often characterized by small leaves with a mean leaf area about 20–40 cm^2^ (Rajkumar et al., 2010; Xie et al., 2015; Tan et al., 2018). However, single CSS varieties can have also large (40–60 cm^2^) and extralarge (>60 cm^2^) leaves, as observed in different Chinese regions (Wang and Tang, 2012).

### Efficiency of Simple Sequence Repeat and Start Codon Targeted Markers for Tea Germplasm Discrimination

In numerous studies, SSRs served as excellent markers in genetic analyses of tea plants (Fang et al., 2012; Taniguchi et al., 2014; Zhao et al., 2014; Wambulwa et al., 2016; Wang et al., 2016; Liu et al., 2018; Meegahakumbura et al., 2018; Lee et al., 2019). Due to their codominant inheritance, polymorphism, ease and reliability of evaluation, SSRs may have significant advantages over dominant anonymous markers (e.g., SCoT, RAPD, or AFLP) for many applications in population genetics. However, for polyploids, such as several tea mutants, allele dosage of SSRs cannot be readily determined reliably (Pfeiffer et al., 2011). In this case, dominant markers such as SCoT markers might have an advantage because they avoid problems with dosage uncertainty (Dufresne et al., 2014). SCoT markers have been successfully applied for diversity analysis and fingerprinting in many crops. In tea, SCoT markers have also been used in three studies for genetic diversity analysis (Lin et al., 2018; Xu et al., 2019; Chaeikar et al., 2020). Therefore, SCoT markers were selected to compare their efficiency with selected SSR marker in 18 accessions with higher genome size along with 23 accessions of clonal and mutant origin.

Despite of the occurrence of polyploid accessions within the dataset, no multibanding was observed for the SSRs. Nevertheless, the average number of detected alleles was with *Na* = 4.79 for SSR dataset remarkably higher than for the SCoT dataset with *Na* = 1.58. The calculated diversity was also higher based on the SSR dataset (*He* = 0.53) than based on the SCoT dataset (*H* = 0.23). The SCoT markers in the selected tea accession showed 66.7% polymorphism, which was lower than in other tea studies. For example Lin et al. (2018) reported 93.15% polymorphism in 55 tea accessions, Xu et al. (2019) reported 83.7% of polymorphism in 18 tea clones, and Chaeikar et al. (2020) identified 73.4% polymorphic alleles for 9 tea accessions. However, particularly in the two first studies, the number of SCoT markers used were remarkably higher than in our study. In contrast, it was obvious that the number of SCoT markers used were not sufficient to discriminate the selected tea accessions in our study. Based on the SCoT markers (*PD* = 0.71) only 20 individuals showed different allelic pattern whereas based on the SSR markers (*PD* = 0.74) all 43 accessions were distinguished. This result demonstrates that SSR markers, despite of the occurrence of polyploids in the tea collection, are more precise and reliable and fewer loci are required than for dominant SCoT markers.

### Comparing Genetic Distances Among Tea Genotypes Based on Simple Sequence Repeat and Start Codon Targeted Markers

Mantel tests showed no correlation between SCoT and SSR data matrixes. This result is in accordance with Zhang Y. et al. (2018) who found weak or no correlation between SCoT and EST-SSR molecular markers. Other researchers analyzing tea collections also did not find any correlation between the different multilocus DNA-markers (Falakro and Khiavi, 2020). The low correlation between these markers probably reflects that these markers target distinct genomic regions with repeat and/or unique sequences. These may have evolved differentially or may have been preserved during artificial selection.

### Genetic Diversity, Genetic Structure, and Relationships in Tea Germplasm Collection Based on Simple Sequence Repeat Genetic Data

Our results on SSR marker efficiency are largely consistent with those of Wang et al. (2016). We observed the highest *Ho* for TM337 and TM589 and the lowest *Ho* for TM514, which is in accordance with Wang et al. (2016). In terms of the average allele numbers, we observed higher number of alleles for most SSR marker (e.g., TM352; T447) than in the study of Wang et al. (2016), possibly due to the broader genetic background of accessions in the Russian tea collection. Many private SSR alleles were detected in our study that can be useful for fingerprinting and accurate cultivar identification, which in turn is the basis for commercial certification and protection of tea cultivars. SSR analysis of the Russian collection confirmed a complicated genetic background and high genetic diversity, despite the claim by some researchers (Yao et al., 2012) that allele number and genetic diversity of tea accessions decrease significantly with increasing distance from the center of origin.

Our STRUCTURE results showed a high level of genetic admixture in many accessions, confirming a mixed origin of the germplasm collection during the breeding process. From the domestication history of tea in the Caucasus, Indian tea plants (CSA) were introduced here together with Chinese plants (CSS). The first cluster included 31 accessions, mainly representing the most cold-tolerant genotypes in our collection. The second cluster consisted mainly of hybrids, probably derived from Quimen landraces, but also from several mutant forms and clonal varieties. It is believed that these two clusters contained mainly CSS tea cultivars, which is in accordance with other studies that also clustered CSS and CSA in separate groups (Huang et al., 2014). CSS have a broad geographic origin and therefore originate from complex natural environments. This may have led to the occurrence of more adaptive mutations, which is why these accessions have high genetic diversity (An et al., 2020). In contrast, cluster 3 probably contained mostly CSA × CSS hybrids. Many cultivars in cluster 3, including the best national cultivar “Kolkhida,” show typical phenotypic characteristics of CSA, such as large, thin, soft, and wrinkled leaves of light green color. CSA were also introduced in the Caucasus, but most CSA accessions are distributed in tropical regions and have lower cold resistance (Hao et al., 2018; An et al., 2020). For this reason, most of these CSA accessions introduced to the Caucasus are thought to have likely died and only CSS × CSA hybrids have survived.

On the other hand, many genotypes in cluster 3 derived from “Kolkhida” have higher ploidy level. This suggests that leaf size is not only due to CSA type, but also due to ploidy level or earlier breeding selection. This is consistent with other studies that showed the classification of tea varieties into two groups after STRUCTURE analysis: (1) tea with large leaves and (2) tea with medium/small leaves (Liu et al., 2017; Yu et al., 2020). Similar grouping by leaf size was confirmed in our study using the phylogenetic tree. In general, large and extra-large genotypes predominate in the Russian tea collection. The reason could be that for decades two main criteria for phenotypic selection of tea were applied, namely, leaf length > 13 cm and leaf width > 6 cm. These can be derived from both large-leaf CSS cultivars and CSS × CSA hybrids. Unfortunately, there are still no efficient DNA markers for reliable discrimination of CSA and CSS.

## Conclusion

Remarkable variability in genome size was observed in 43 cultivars and mutants, and the increase in genome size was confirmed in 18 mutants within this collection. Despite the presence of polyploid tea genotypes, our study showed higher efficiency of SSR markers than SCoT markers. Based on the SSR markers, the phylogenetic relationships of 106 tea plant accessions were then successfully revealed, which provided valuable information for understanding the phylogeny and genetic origin of these accessions in Russian tea germplasm.

The STRUCTURE analysis grouped the accessions into three distinct genetic clusters. Greater genetic variation was observed within genetic clusters than between clusters, indicating low genetic variation between collections. Nevertheless, the most northerly grown tea genotypes from Adygea had the greatest genetic distance from accessions compared with those from the other regions. These showed close genetic relationship and consisted mainly of several small- and medium-leaved cultivars that could be donors of the cold tolerance trait.

Close genetic relationships were also found between many cultivars with particularly large leaves and mutant forms. In particular, the close relationship of many large-leaved mutants with the cultivar “Kolkhida” confirmed the ancestry of many locally evolved genotypes.

Thus, the results of our study contribute to the development of future strategies for tea germplasm conservation and modern tea breeding programs.

## Data Availability Statement

The original contributions presented in the study are included in the article/Supplementary Material, further inquiries can be directed to the corresponding author.

## Author Contributions

LS contributed to conceptualization, investigation, data analysis, visualization, draft manuscript, and funding acquisition. AM, RS, NK, AS, and KM contributed to investigation and data analysis. SL, JZ, and LM handled conceptualization, data analysis, manuscript-critical revisions, and editing. MG, VM, AR, and EP handled genetic resources, phenotyping, formal analysis, and administration. HF and M-VH were responsible for conceptualization, resources, and administration. SR handled conceptualization, supervision, data analysis, visualization, software, manuscript-critical revisions, and editing. All authors contributed to the article and approved the submitted version.

## Conflict of Interest

The authors declare that the research was conducted in the absence of any commercial or financial relationships that could be construed as a potential conflict of interest.

## Publisher’s Note

All claims expressed in this article are solely those of the authors and do not necessarily represent those of their affiliated organizations, or those of the publisher, the editors and the reviewers. Any product that may be evaluated in this article, or claim that may be made by its manufacturer, is not guaranteed or endorsed by the publisher.

## Footnotes

1
https://cgspace.cgiar.org/handle/10568/72781

2
https://irscope.shinyapps.io/iMEC/

## Supplementary Material

Supplementary Table 1 List of collection and phenotypical evaluation.

Supplementary Table 2 SSR and SCoT primer sequences.
